# Should we reconsider iron administration based on prevailing ferritin and hepcidin concentrations?

**DOI:** 10.1007/s10157-012-0694-3

**Published:** 2012-09-29

**Authors:** Takeshi Nakanishi, Takahiro Kuragano, Shoji Kaibe, Yasuyuki Nagasawa, Yukiko Hasuike

**Affiliations:** Division of Kidney and Dialysis, Department of Internal Medicine, Hyogo College of Medicine, 1-1 Mukogawa-cho, Nishinomiya, 663-8501 Japan

**Keywords:** Hepcidin, Iron, Renal anemia, Erythropoiesis stimulating agents, Ferritin

## Abstract

The results of recent randomized, controlled trials in patients with chronic kidney disease and anemia have suggested that hyporesponsiveness to erythropoiesis stimulating agents (ESA) is a significant predictor of poor patient outcomes. Functional iron deficiency (FID) is the most common cause of suboptimal ESA response, and intravenous iron administration (IVFe) efficiently raises hemoglobin (Hb) concentrations even under the condition of FID. Consequently, renal anemia correction has conceptually shifted from ‘higher Hb values with high ESA doses’ to ‘prevention of ESA hyporesponsiveness with IVFe’. The discovery of hepcidin has profoundly changed our understanding of the place of FID in renal anemia therapy. Hepcidin reduces the abundance of iron transport proteins which facilitate iron absorption from the gut and iron mobilization from macrophages. Serum hepcidin is mainly modulated by iron stores, as is serum ferritin. High hepcidin or ferritin levels block intestinal iron absorption and iron recycling in macrophages and decrease iron availability for erythropoiesis, leading to FID. Iron administration, especially IVFe, increases hepcidin levels and concomitantly inhibits iron supply to erythroid cells. This in turn could lead to a vicious circle, exacerbating FID and increasing iron demand. Therefore, physicians should be cautious with unrestricted IVFe to chronic kidney disease patients with FID.

## Introduction

Recently, several large cohort studies investigating renal anemia therapy have highlighted the biologically plausible, but erroneous assumption that the normalization of hemoglobin (Hb) iron should attenuate cardiovascular disease risks and lead to a decline in the mortality rate of patients with chronic kidney disease (CKD), both before and after the initiation of maintenance hemodialysis (MHD) treatment [1–4]. Erythropoiesis stimulating agent (ESA) treatment decisions and guidelines based on the questionable assumption that Hb should be normalized or nearly normalized in the majority of CKD patients need to be reconsidered [5]. The development of safe and effective strategies aimed at obtaining better patient survival remains a challenge. In recent years, high-dose intravenous (IV) iron supplementation has become the standard of care; however, there are concerns as to whether this is the right approach.

Recent studies on the mechanisms involved in iron metabolism have revealed that hepcidin is a master regulator of systemic iron availability [6, 7]. To maintain iron homeostasis, hepcidin tightly controls duodenal iron absorption and iron recycling from senescent erythrocytes by tissue macrophages. Hepcidin is the principal hormone responsible for the physiological regulation of iron balance as well as its control in a variety of pathologic conditions, including the anemia of chronic disease (ACD).

In this review, we address the mechanisms whereby pharmacological iron supplementation, especially via the IV route, may reduce the body’s capacity to absorb iron from the gut and to reutilize iron from endogenous sources [8], with particular focus on the importance of hepcidin in this process.

## ESA hyporesponsiveness

Although normal or near-normal Hb levels in CKD patients were associated with reduced mortality in many observational studies [9–11], recent evidence from randomized clinical trials does not support a beneficial effect of Hb normalization on survival. This was demonstrated by a meta-analysis which showed that targeting higher Hb concentrations with ESA treatment was associated with increased all-cause mortality and cardiovascular events [12]. Several post hoc analyses of these studies led to the hypothesis that hyporesponsiveness to ESA (i.e., high doses of ESA required to correct Hb levels) was a major determinant of negative outcomes in patients with CKD [12–14].

Among the randomized clinical trials, the Normal Hematocrit Cardiac Trial (NHCT), which to date remains the largest randomized ESA trial conducted in hemodialysis patients, showed that targeting a normal hematocrit was associated with a 1.3-fold increased risk of mortality or nonfatal myocardial infarction [1]. Nevertheless, patient survival was best among those who had achieved the highest hematocrit values [13, 15]. A subsequent reanalysis of the data demonstrated that ESA hyporesponsiveness was a significant predictor of 1-year mortality in the high hematocrit group [15].

As it is generally accepted that iron deficiency is the most common cause of ESA hyporesponsiveness, the recent European Renal Best Practice (ERBP) recommendations and other comments on these studies recognized iron supplementation as an essential part of anemia treatment [16, 17]. The ERBP group’s suggestion is to use iron replacement first in any CKD patient who is proven or likely to be iron-deficient, and only when the iron stores have been replenished should ESA therapy be initiated.

However, the results of the NHCT suggest that generous iron administration contributes to increased risk of death [1]. Iron supplementation, an ancillary treatment of renal anemia therapy, was used together with ESA therapy in the NHCT study. As large amounts of iron are necessary to allow an increase in Hb production, intravenous iron was administered more frequently to patients in the normal than in the low hematocrit group. The mortality risk was greatly increased in those who were treated with intravenous iron 6 months before death or censoring (odds ratio 2.4; *P* < 0.001) [1].

This is one among several reasons why we should focus equally on the potential harms of over-utilization of IV iron and of ESAs. There is no evidence that it is safer in patients on MHD with evidence of inflammation to achieve a given Hb target with less ESA and more iron than to achieve the same Hb target with more ESA and less iron. This is probably true even for patients who only have microinflammation and/or elevated serum cytokine levels.

## Functional iron deficiency

The most common condition known to cause incomplete ESA response is decreased iron availability, including absolute and functional iron deficiency (FID) [18]. The close interdependence of iron and ESA administration in the successful treatment of renal anemia is clearly established [19–21]. The best proof of iron-deficient erythropoiesis is the response to IV iron [22], which has become standard of care to optimize ESA efficacy [23]. Iron loss is common in hemodialysis patients. It occurs because of blood loss into dialysis lines and filters, frequent laboratory testing, and gastrointestinal bleeding, to name but a few causes. Many patients who have borderline low iron stores at the start of ESA therapy develop absolute iron deficiency as these stores become depleted during the production of new red blood cells. Others with adequate or even excessive iron stores may develop FID. The latter occurs when sufficient amounts of iron cannot be released from its reserves, mostly the reticuloendothelial system (RES) to satisfy the increased demand of the bone marrow during ESA-induced erythropoiesis, as is often the case in ACD [20, 21]. FID is the most common cause of suboptimal ESA response, leading physicians to use IV iron to improve its availability [24, 25].

The previous belief that IV iron therapy would become progressively inefficient with increasing serum pretreatment ferritin levels, and be practically useless with pretreatment ferritins >500 ng/ml [26] has been contradicted by a recent trial, the Dialysis Patients’ Response to IV iron with Elevated ferritin (DRIVE) study [27]. The authors of this study demonstrated that IV ferric gluconate administration was superior to no iron treatment in improving hemoglobin levels in anemic hemodialysis patients with ferritin levels of 500–1200 ng/ml and transferring saturation (TSAT) >25 %. The conclusion from observations such as this one is that intravenous iron administration can effectively raise Hb even in patients with elevated iron stores.

Following the report of the DRIVE study, there has been a tendency towards increasing the upper limit of serum ferritin levels. However, it must be emphasized that there is no proof at present that pushing up Hb levels with excessive iron doses improves the vital prognosis of MHD patients. It could even do the opposite.

## Transfer of intravenous iron to erythroid cells

We do not completely understand the exact mechanism involved in the improvement of Hb levels or ESA response subsequent to IV iron administration. Based on previous pharmacokinetic studies, however, one can speculate how parenteral iron may be utilized for erythropoiesis. The pharmacokinetics of parenteral iron sucrose or iron–polysaccharide complexes have been assessed using positron emission tomography [28, 29]. These studies demonstrated that non-saturation of the transport system allows iron transfer from the blood to the bone marrow, indicating the presence of a large interstitial transport pool. Similar observations were reported in previous ferrokinetic studies using radiolabeled iron (^59^Fe) where time-dependent accumulation of ^59^Fe was detected over the sacrum, a site of hematopoietic marrow [30].

Erythroid precursors have an extremely high iron requirement, especially during Hb synthesis. Although it was believed that the major pathway of iron supplementation for erythropoiesis is the transferrin receptor 1 (TfR1) pathway in erythroid cells, another route of iron supplementation to erythroid cells has also been demonstrated [31, 32]. Bone marrow macrophages support the development of erythroid progenitors under transferrin (Tf)-free conditions by delivering essential iron for erythropoiesis in the form of metabolizable ferritin [33]. Thus, iron can be supplied to erythroid cells for hemoglobin synthesis using transferrin from plasma as well as ferritin from bone marrow macrophages.

Recently, Coulon et al. [34] demonstrated that TfR1 plays an important role in erythropoiesis, besides the transport of Tf-bound iron into erythroid progenitors. TfR1 engagement by either polymeric immunoglobulin (Ig)A1 (pIgA1) or diferric Tf (Fe_2_-Tf) increased cell sensitivity to erythropoietin by inducing activation of mitogen-activated protein kinase and phosphatidylinositol 3-kinase signaling pathways. Fe_2_-Tf could act together with pIgA1 on TfR1 to promote robust erythropoiesis in both physiological and pathological situations, which may be relevant to IV iron administration. Further studies are necessary to support and clarify these mechanisms.

## Anemia of chronic disease

The anemia of CKD shares some of the characteristics of ACD, although decreased erythropoietin production secondary to chronic kidney failure, as well as the anti-proliferative effects of accumulating uremic toxins, significantly contribute to the pathogenesis of the former [35, 36]. In patients with end-stage renal disease, higher levels of proinflammatory cytokines such as tumor necrosis factor alpha (TNFα) and interleukin-6 (IL-6) have been consistently observed and are thought to contribute to ACD [37, 38].

A hallmark of ACD is disturbed iron homeostasis, with increased import, decreased export and retention of iron within cells of the RES. This leads to a maldistribution of iron from the circulation into storage sites of the RES, subsequent limited iron availability for erythroid progenitor cells, and iron-restricted erythropoiesis. In mouse models or cultured cells that are exposed to proinflammatory agents such as lipopolysaccharide, IL-1 and TNFα there is upregulation of the expression of divalent metal transporter 1 (DMT1) with increased iron uptake by activated macrophages [39]. These proinflammatory stimuli also induce the retention of iron in macrophages by down-regulating the expression of ferroportin (FPN), thereby blocking the cellular release of iron. Similar findings were made in human umbilical endothelial cells [40].

The proinflammatory cytokine-related mechanisms, which play a major role in the reduction of iron transfer to the bone marrow, include not only an impairment of iron release and transport from the RES (storage tissue) but also a decrease in iron absorption from the gut. One controversial point is that the concentration of proinflammatory cytokines required to affect these iron transport proteins is considerably higher than the serum levels that have are generally observed in patients on MHD. In contrast to the cytokines, hepcidin appears to affect the expression of iron transport proteins within the range of serum concentrations of healthy subjects and patients with ACD [8]. Therefore, the discovery of hepcidin and its function had a tremendous impact on our understanding of normal and pathologic iron metabolism and related disorders, including ACD.

## Hepcidin affects iron transport proteins

**Fig. 1:**
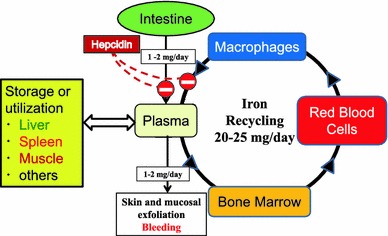
Iron recycling and absorption is blocked by hepcidin. Iron recycled from the continuous breakdown of hemoglobin in senescent red cells by reticuloendothelial macrophages is essential to meet the requirements of erythropoiesis (20–30 mg/day). Absorption of dietary iron (1–2 mg/day) is tightly regulated depending on body needs, and just balanced against iron loss. There is no physiological mean by which excess body iron is excreted. Hepcidin is an iron regulatory hormone that maintains systemic iron homeostasis. It is made by the liver and secreted into the blood stream, where it causes iron transport proteins, ferroportin and divalent metal transporter 1, to be degraded. As a result, hepcidin reduces gastrointestinal iron absorption and macrophage-mediated iron recycling

Following its discovery >10 years ago, hepcidin has progressively been recognized as a central player in the regulation of systemic and local iron homeostasis [8, 41, 42]. This small peptide hormone produced by the liver inhibits iron efflux from cells by interacting with the iron export protein, FPN, especially in iron-recycling macrophages, and the iron import protein, DMT1, in duodenal enterocytes. The binding of hepcidin to FPN results in the internalization and lysosomal degradation of FPN, which inhibits iron release by macrophages [43]. In addition, hepcidin also degrades DMT1 via the ubiquitin-dependent proteasome pathway, which results in the reduction of intestinal iron absorption [44]. Hepcidin treatment reduces the abundance of these iron transport proteins in a dose-dependent manner (Fig. 1). While a high concentration of hepcidin will acutely decrease the expression of iron transport proteins, a lower concentration may affect FPN and DMT1 abundance more slowly. In the clinical setting, even relatively low concentrations of hepcidin may exert a prolonged effect on iron metabolism with continuous exposure of cells to hepcidin, resulting in a consistent down-regulation of FPN and DMT1 [8].

## Hepcidin is exclusively dependent on ferritin, and not superior to ferritin for monitoring iron need

As observed in a previous study by our group, serum ferritin has the highest predictive value for serum hepcidin levels, as confirmed by several recent studies [45–47]. The relationship between serum hepcidin and inflammatory markers is less clear in patients with CKD, although hepcidin expression was initially found to be induced by IL-6 in inflammatory conditions [48]. In our study in MHD patients with high-sensitivity C-reactive protein (hs-CRP) levels <0.3 mg/dl, serum IL-6 and TNFα levels were significantly higher than in healthy volunteers, but these concentrations were not correlated with hepcidin. Only in the group of patients with higher hs-CRP levels (≥0.3 mg/dl) were both IL-6 and ferritin significant predictors of hepcidin by multivariate analysis. We therefore assume that the expression of hepcidin-25 is principally associated with ferritin in stable MHD patients without apparent inflammatory disease [8]. Thus, the serum hepcidin level is principally modulated by iron stores, which in turn are generally reflected by the serum ferritin level [49]. The relationship between serum ferritin and iron storage has been investigated, and the expression of ferritin was exclusively dependent on iron, even in patients with ACD [49].

**Fig. 2:**
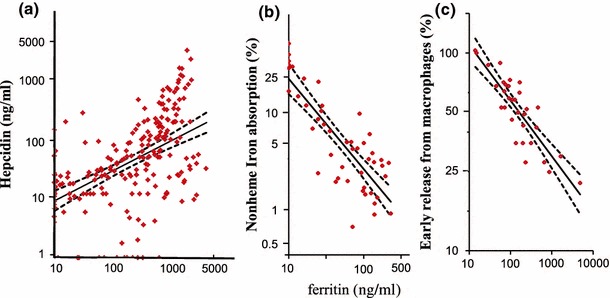
Correlation between serum ferritin and hepcidin levels (**a**), percent nonheme iron absorption (**b**), and percent early iron release from macrophages (**c**). **a** Serum ferritin levels are significantly correlated with serum hepcidin levels in both healthy volunteers and MHD patients (recalculated from the relationships depicted in the study by Kuragano et al. [8, 45]) (log[hepcidin] = 0.72 × log[ferritin (ng/ml)] − 0.17; *r* = 0.64; *P* < 0.01). **b** A highly significant inverse correlation is observed between serum ferritin and the percentage of absorbed nonheme iron in healthy volunteers (log[nonheme iron absorption (%)] = −0.84 × log[ferritin (ng/ml)] + 2.07; *r* = 0.82; *P* < 0.001 [8, 54]). **c** Serum ferritin levels are significantly correlated with early iron release derived from senescent red blood cells of the reticuloendothelial system in healthy subjects and in patients with iron deficiency, inflammation, marrow aplasia, and hyperplastic erythropoiesis, respectively. Patients with hemochromatosis have been excluded from the analysis because they may have defects in hepcidin synthesis. The calculation of early release of radiolabeled-iron from the reticuloendothelial system is based on the rate of ^55^Fe transferrin clearance and the reappearance of transferrin ^59^Fe derived from radiolabeled heat-damaged red blood cells. (log[early iron release(%)] = −0.28 × log[ferritin (ng/ml)] +2.32; *r* = 0.86; *P* < 0.001; [58])

Recent reports have confirmed that iron stores are the major determinant of serum hepcidin levels as well as iron mobilization. In rats and humans with ACD, serum hepcidin concentrations are elevated, and this is paralleled by reduced duodenal and macrophage expression of FPN. The coexistence of ACD and iron deficiency anemia (IDA) results in a smaller increase in hepcidin expression. Correspondingly, individuals with ACD/IDA have significantly lower hepcidin levels than patients with ACD alone. Moreover, ACD/IDA patients, in contrast to ACD subjects, were found to be able to absorb dietary iron from the gut and mobilize iron from macrophages. These data again demonstrate that circulating hepcidin levels are mainly dependent on iron stores and perturbed iron traffic, even in the presence of ACD [50]. These observations have been confirmed in recent clinical studies and mouse models of chronic inflammation [50–52].

For managing iron therapy in MHD patients being treated with ESA, it has been hypothesized that measuring serum levels of hepcidin may be useful as an additional tool for predicting and monitoring the need for iron supplementation. However, the recent clinical observations demonstrated that it could not provide an advantage over established markers of iron status, ferritin and TSAT [47, 53].

## Hepcidin and iron regulation in the intestine and macrophages

As mentioned above, serum hepcidin levels were found to be tightly linked to circulating ferritin levels in both healthy volunteers and MHD patients [8, 45]. To estimate the relationship between serum hepcidin levels and iron absorption serum ferritin may be used as a surrogate for hepcidin, as depicted in Fig. 2a. A highly significant inverse correlation between iron stores, as reflected by serum ferritin, and the absorption of nonheme iron was consistently found in healthy subjects and MHD patients [54–57] (Fig. 2b). As the serum ferritin decreased with iron deficiency (<100 ng/ml), a 10-fold rise in nonheme iron absorption occurred [54]. This indicates that depletion of body iron stores accelerates the dietary absorption of non-heme iron [54]. This effect is probably due to the control of iron absorption by hepcidin.

A similar relationship between body iron stores or serum ferritin levels and iron egress from macrophages has been observed [58]. Hepcidin also appears to play a fundamental role in iron homeostasis in the RES. Iron recycles from senescent erythrocytes to macrophages and back to circulation (approximately 20–25 mg/day), resulting in an iron supply to erythroid cells which is far greater than that provided by duodenal absorption (1–2 mg/day). Erythrocyte iron processing by the RES was studied after intravenous injection of ^59^Fe-labeled heat-damaged red blood cells and ^55^Fe-labeled transferrin to calculate the early release of ^59^Fe by the RES [58]. Interestingly, there was a significant negative correlation between the percentage of early iron release by macrophages and serum ferritin (Fig. 2c). This has led to the conclusion that storage iron tightly modulates the release of iron into the circulation from the intestine and from macrophages under the control of hepcidin.

Recently, factors affecting erythrocyte iron incorporation were analyzed in anemic pediatric patients treated with oral iron. It was concluded that hepcidin powerfully controlled the utilization of dietary iron by erythrocytes, as serum hepcidin was inversely correlated with RBC iron incorporation [59].

**Fig. 3:**
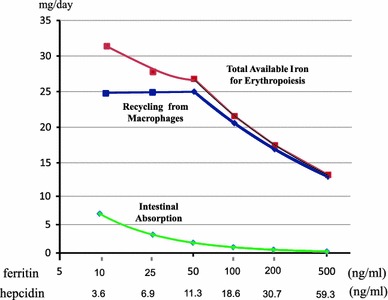
Estimated serum hepcidin levels, intestinal iron absorption, iron release from macrophages, and total available iron available for erythropoiesis. These parameters vary according to serum ferritin levels. Based on the relationship between serum ferritin and hepcidin levels, percent nonheme iron absorption, and percent early iron release from macrophages (see Fig. 2), we can estimate the total iron available for erythropoiesis. For these calculations, we assume that iron absorption is 1 mg/day and iron release by macrophages 20 mg/day for a serum ferritin level of 100 ng/ml, and a maximal amount of iron recycling by macrophages of 25 mg/day. Based on these calculations, the estimated amount of iron available for erythropoiesis decreases with increasing concentrations of serum ferritin

Although we do not understand the extent to which hepcidin blocks iron transport in macrophages and duodenal epithelial cells, we can estimate intestinal iron absorption and iron release from macrophages based on the reported associations between serum ferritin, serum hepcidin, percent nonheme iron absorption, and percent early iron release from macrophages. We can thus assume that iron absorption amounts to 1 mg/day and iron release from macrophages to 20 mg/day when the serum ferritin level is 100 ng/ml and maximal iron recycling in macrophages is 25 mg/day. Consequently, as shown in Fig. 3, the estimated relative amount of iron available for erythropoiesis decreases as serum ferritin increases. The concentration of hepcidin, which can be estimated from the ferritin–hepcidin relationship, is somewhat lower than the half maximal inhibitory concentration of hepcidin observed in cell culture models but may be effective after long-term exposure as is the case under clinical conditions [45, 60].

## Iron usage in Japan and worldwide

In the prospective study of the hemodialysis patient cohort of the Japan Dialysis Outcomes and Practice Patterns Study (DOPPS) in 2007, mean Hb and serum ferritin levels were 10.38 g/dl and 224 ng/ml, respectively, and the percentage of patients with ferritin levels <100 ng/ml was 41.3 % [61]. Of note, the 47.2 % of patients with Hb ≥11 g/dl had ferritin levels <100 ng/ml, and only 40.6 % of them received IV iron. These observations suggested that a substantial percentage of patients could maintain Hb levels >11.0 g/dl without iron supplementation, owing to intestinal iron absorption. Therefore, the amount of iron absorbed from the intestine could compensate for that lost in the blood of these patients.

From the 2010 DOPPS Annual Report (http://www.dopps.org/annualreport/index.htm), mean serum ferritin levels were >400 ng/ml in patients from all countries except Japan. In the United States, which represented the majority of patients included in the DOPPS, mean serum ferritin levels were >550 ng/ml, and 73.7 % of these patients were receiving IV iron. As the serum ferritin level is associated with hepcidin, in patients with serum ferritin levels >500 ng/ml iron absorption and iron recycling in macrophages could be minimal. In these situations, less intestinal iron absorption compelled physicians to use IV iron to maintain iron balance, which in turn led to a further increase in storage iron. From these analyses we deduce that the difference in ferritin and hence in hepcidin levels between Japan and the other DOPPS countries is probably linked to the differences in the amount of iron administered.

A possible caveat of this supposition is that there was also a difference in achieved Hb levels between dialysis patients in Japan and those in the other DOPPS countries. However, the Japanese Society for Dialysis Therapy explained the difference between Japan and other countries by timing of blood collection and patient position at blood collection. Blood sampling for studies of Hb levels is performed at the beginning of the week in Japan, whereas it is generally performed on the middle day of the week in the other countries [62]. This difference in sampling time could affect the rate of weight gain and plasma volume. In addition, the supine position at blood collection may further decrease the Hb values in Japan, whereas the majority of patients in the other countries undergo MHD in a sitting position on a chair-bed. Further investigation is needed to clarify the cause underlying the differences in ferritin and Hb levels between dialysis patients in Japan and other countries.

## Conclusion

It has long been recognized that the most common cause of incomplete ESA response is limited iron availability, and that iron supplementation may improve the response to ESA. Increased blood loss is inherent to the condition of hemodialysis patients. Therefore, the use of IV iron is frequently indicated to maintain iron balance. However, there is no convincing evidence that IV iron supply improves patient survival although FID is a major cause of ESA hyporesponsiveness which itself is tightly associated with the poor outcomes of anemic patients with CKD. The discovery of hepcidin has considerably improved our understanding of the regulation of iron metabolism and related disorders. It has also profoundly changed our view of iron supplementation. When hepcidin concentrations are high, FPN is internalized, iron is trapped in macrophages, DMT1 is degraded, and iron absorption in the intestine is minimal. Based on the close correlation between ferritin and hepcidin, iron administration should increase hepcidin levels, which in turn should not only reduce the release of iron and its transport from the RES (storage tissues) but also decrease iron absorption from the gut. These effects are consistent with findings in ACD patients as well as in those with FID. We suggest that physicians be cautious in prescribing IV iron in patients with FID, even if the immediate effect is an improvement in the anemia management of iron-replete MHD patients. No long-term safety data exist with respect to the effects of prolonged IV iron therapy on hard patient outcomes. Large randomized prospective cohort studies are needed to answer the question of whether a better MHD patient survival is achieved with less ESA and more IV iron or more ESA and less IV iron.
